# Effect of B-NIPOx in Experimental *Trypanosoma cruzi* Infection in Mice

**DOI:** 10.3390/ijms24010333

**Published:** 2022-12-25

**Authors:** Albany Reséndiz-Mora, Giovanna Barrera-Aveleida, Anahi Sotelo-Rodríguez, Iván Galarce-Sosa, Irene Nevárez-Lechuga, Juan Carlos Santiago-Hernández, Benjamín Nogueda-Torres, Sergio Meza-Toledo, Saúl Gómez-Manzo, Isabel Wong-Baeza, Isabel Baeza, Carlos Wong-Baeza

**Affiliations:** 1 Laboratorio de Biomembranas, Departamento de Bioquímica, Escuela Nacional de Ciencias Biológicas, Instituto Politécnico Nacional, Mexico City 11340, Mexico; 2 Laboratorio de Enzimología, Departamento de Bioquímica, Escuela Nacional de Ciencias Biológicas, Instituto Politécnico Nacional, Mexico City 11340, Mexico; 3 Laboratorio de Helmintología, Departamento de Parasitología, Escuela Nacional de Ciencias Biológicas, Instituto Politécnico Nacional, Mexico City 11340, Mexico; 4 Laboratorio de Quimioterapia Experimental, Departamento de Bioquímica, Escuela Nacional de Ciencias Biológicas, Instituto Politécnico Nacional, Mexico City 11340, Mexico; 5 Laboratorio de Bioquímica Genética, Instituto Nacional de Pediatría, Secretaría de Salud, Mexico City 04530, Mexico; 6 Laboratorio de Inmunología Molecular II, Departamento de Inmunología, Escuela Nacional de Ciencias Biológicas, Instituto Politécnico Nacional, Mexico City 11340, Mexico

**Keywords:** Chagas disease, treatment, *Trypanosoma cruzi*, metabolism inhibition, benzyl ester of N-isopropyl oxamic acid

## Abstract

Chagas disease is caused by *Trypanosoma cruzi* and represents a major public health problem, which is endemic in Latin America and emerging in the rest of the world. The two drugs that are currently available for its treatment, Benznidazole and Nifurtimox, are partially effective in the chronic phase of the disease. In this study, we designed and synthesized the benzyl ester of N-isopropyl oxamic acid (B-NIPOx), which is a non-polar molecule that crosses cell membranes. B-NIPOx is cleaved inside the parasite by carboxylesterases, releasing benzyl alcohol (a molecule with antimicrobial activity), and NIPOx, which is an inhibitor of α-hydroxy acid dehydrogenase isozyme II (HADH-II), a key enzyme in *T. cruzi* metabolism. We evaluated B-NIPOx cytotoxicity, its toxicity in mice, and its inhibitory activity on purified HADH-II and on *T. cruzi* homogenates. We then evaluated the trypanocidal activity of B-NIPOx in vitro and in vivo and its effect in the intestine of *T. cruzi*-infected mice. We found that B-NIPOx had higher trypanocidal activity on epimastigotes and trypomastigotes than Benznidazole and Nifurtimox, that it was more effective to reduce blood parasitemia and amastigote nests in infected mice, and that, in contrast to the reference drugs, it prevented the development of Chagasic enteropathy.

## 1. Introduction

Chagas disease, or American Trypanosomiasis, is the most serious endemic parasitic disease in Latin America, with an estimated morbidity burden that is between 5 and 10 times higher than the burden of malaria [1]. Since it affects 6 to 7 million people worldwide, with around 7500 deaths per year, it is considered a public health problem [2]. In the USA and Europe, which are non-endemic areas, around 300,000 and 97,556 infected individuals are estimated, respectively [3]. This disease is caused by the flagellated protozoan parasite *Trypanosoma cruzi*, and it is mainly transmitted to mammals through the feces or urine of triatomine insects (its main vector) [4]. Other causes of transmission are blood transfusions, organ transplantations, the ingestion of contaminated food, and transplacental transmission [5,6]. The infection occurs when trypomastigotes, the non-replicative flagellated form of *T. cruzi*, enter the bloodstream and infect several nucleated cell types. The trypomastigotes mature into amastigotes and multiply intracellularly until they differentiate again into trypomastigotes, which cause cell lysis. These trypomastigotes are released into the circulation and spread through the blood, infecting mainly the spleen, the liver and the heart muscle [7].

Chagas disease has an acute and a chronic phase. The acute phase is characterized by a high degree of parasitemia, usually without symptoms. However, prolonged fever, headaches, myalgia, hepatomegaly and splenomegaly are observed in some cases. After 4 to 8 weeks, the disease progresses into the chronic phase, with decreased parasitemia and symptom improvement, but with positive serology [8]. Between 60 and 70% of the patients in the chronic phase remain without symptoms. However, after 10 years, some asymptomatic patients can present neurological, digestive (esophagopathy and colopathy), cardiac or cardiac–digestive clinical conditions [8]. The most evident digestive conditions in Chagas disease are the megaesophagus and megacolon syndromes, which are characterized by hypertrophy of muscle layers, motor dysfunction of segments of the colon and constipation (Orozco et al., 2022). Denervation of the nervous system that regulates the motor functions of the digestive tract also occurs, resulting in dysphagia, hypomotility and distension, which can cause swallowing problems, regurgitation and constipation [8].

Currently, Benznidazole and Nifurtimox are the only two drugs that are available for the treatment of Chagas disease. However, they have toxic effects and induce parasite resistance, and they are not very effective in chronic patients [9]. Nifurtimox has secondary effects such as hypersensitivity reactions, anorexia, vomiting, polyneuritis and depression, therefor Benznidazole is the most commonly prescribed drug for the treatment of children and adults [10], although its side effects include hepatitis, peripheral polyneuropathy, digestive intolerance and anorexia [9,11]. The curative efficacy of Benznidazole in the acute phase is 60 to 100% but in the chronic phase, it decreases to 8 to 20% [12]. The mechanism of action of Benznidazole is not fully understood but it was proposed that it is activated by NADH-dependent trypanosomal reductases, which form reduced metabolites that damage DNA and inhibit protein synthesis [13,14]. Therefore, the development of new drugs that are safe and effective for the treatment of acute and chronic Chagas disease is indispensable.

Glycolysis is the main metabolic pathway used by *T. cruzi* to obtain its energy [15,16]. In contrast to mammals, in which the reoxidation of NADH + H^+^ is carried out by lactate dehydrogenase, in *T. cruzi*, this reoxidation is carried out by α-hydroxy acid dehydrogenase isozyme II (HADH-II), which mainly reduces α-ketoisocaproate to α-hidroxyisocaproate [17]. HADH-II uses the α-ketoacids α-ketocaproate and α-ketoisocaproate as substrates [17,18]. These α-ketoacids are derived from leucine and isoleucine by transamination, and they also participate in the NADH + H^+^ shuttle system that transfers reducing equivalents from the cytosol to the mitochondria [18]. Therefore, inhibitors that block the activity of HADH-II would inhibit glycolysis and the NADH + H^+^ shuttle system [19], which would significantly decrease the energy available to *T. cruzi* and lead to its death.

In previous works [20,21], we designed and synthesized N-propyl oxamic acid (NPOx) and N-isopropyl oxamic acid (NIPOx), which are structural analogs of the HADH-II substrates α-ketocaproate and α-ketoisocaproate [22]. NPOx and NIPOx are excellent competitive and selective inhibitors of HADH-II, as they have no effect on other dehydrogenases. However, they had low efficacy in in vivo tests because they are polar molecules and the negative charge of their carboxyl groups prevents their passage through cell membranes. Therefore, we synthesized the benzyl ester of NPOx (B-NPOx) [22], which is non-polar and crosses cell membranes. In its esterified form, B-NPOx does not have trypanocidal activity. However, once it reaches the cytoplasm of *T. cruzi*, it is hydrolyzed by parasitic esterases and the released NPOx exerts its inhibitory activity on HADH-II. In this work, we designed and synthesized the benzyl ester of NIPOx (B-NIPOx), because NIPOx is structurally analogous to α-ketoisocaproate, the substrate for which HADH-II has the highest affinity [17]. We evaluated the trypanocidal activity of B-NIPOx in vitro, on epimastigotes and trypomastigotes from four *T. cruzi* strains, as well as in *T. cruzi*-infected mice and it was compared with Benznidazole, Nifurtimox and B-NPOx.

## 2. Results

### 2.1. Characterization of the Benzyl Ester of N-Isopropyl Oxamic Acid (B-NIPOx)

**Figure 1:**
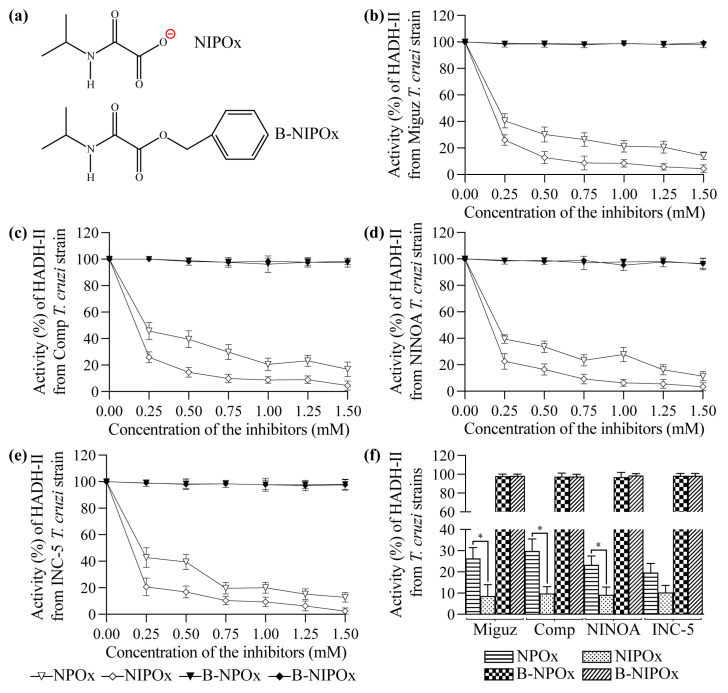
N-isopropyl oxamic acid (NIPOx) is a better inhibitor of the α-hydroxy acid dehydrogenase isozyme II (HADH-II) from *Trypanosoma cruzi* than N-propyl oxamic acid (NPOx). (**a**) Structure of NIPOx (HADH-II inhibitor) and its benzyl ester (B-NIPOx). The inhibition activity of NPOx, NIPOx, B-NPOx and B-NIPOx over the purified HADH-II from (**b**) Miguz, (**c**) Compostela (Comp), (**d**) NINOA and (**e**) INC-5 *T. cruzi* strains were analyzed in the presence of 0.12 mM NADH^+^ + H^+^ and 5 mM α-ketoisocaproate. The inhibitors were evaluated at concentrations of 0.25, 0.50, 0.75, 1.00, 1.25 and 1.50 mM. The HADH-II activity was measured by the changes in the absorption at 340 nm over 5 min and was expressed as a percentage of ΔE 340 nm/min, where the enzymatic activity in absence of inhibitor was considered 100%. (**f**) Asterisks represent statistically significant differences between the HADH-II activity in the presence of 0.75 mM inhibitors (* *p* ≤ 0.05). n = 6. One experiment representative of three is shown.

B-NIPOx was synthesized as described in Materials and Methods. To confirm its structure (Figure 1a), B-NIPOx was analyzed by infrared spectroscopy (IR), proton nuclear magnetic resonance (^1^H-NMR) and carbon-13 nuclear magnetic resonance (^13^C-NMR), where its functional groups were identified, as follows:

IR (ATR): 3279 (N-H), 3086 (C-H, aromatic), 2966, 2879 (C-H methyl), 2937 (C-H methylene), 1768 (C=O, ester), 1668 (C=O, amide), 1350, 1377 (C-H, gem-dimethyl), 709, 789 (aromatic, monosubstituted) cm^−1^.

^1^H-NMR: (500 MHz, CDCl3) δ 1.21 (d, 6H, CH3), 4.10 (sept, 1H, CH), 5.30 (s, 2H, CH2), 7.35–7.44 (m, 5H, H phenyl).

^13^C-NMR: (125 MHz, CDCl3) δ 22.36 (CH3), 41.98 (CH), 68.63 (CH2), 128.72 (Cm phenyl), 128.77 (Co phenyl), 128.90 (Cp phenyl), 134.14 (Cipso phenyl), 157.54 (CO-NH), 159.09 (COO).

These results confirmed that the synthesis of B-NIPOx was successful and since no other groups were detected, it also shows the absence of contaminants in the product.

### 2.2. Evaluation of the Cytotoxicity and Mice Toxicity of B-NIPOx

The Vero, C-33A and HEK293 cell lines presented a viability greater than 97% in the presence of 1, 3, 5 and 10 mM B-NIPOx, and viability between 90 and 95% at 50 and 100 mM, 72 h after the exposure to B-NIPOx, as assessed by the ability of the living cells to reduce alamarBlue. At 24 and 48 h, the viability was greater than 97% in the three cell lines at all concentrations of B-NIPOx. The daily administration of B-NIPOx (1.50–3.00 g/kg) for 50 days did not cause any evident tissue alterations in the mice. The LD_50_ value of B-NIPOx was 2.15 g/Kg of body weight, which is similar to the one reported for B-NPOx (2.00 g/Kg) [22] and indicates that B-NIPOx is not toxic to mice [23].

### 2.3. The Benzyl Ester of N-Isopropyl Oxamic Acid (B-NIPOx) Inhibits the Activity of α-Hydroxy Acid Dehydrogenase Isozyme II in Trypanosoma cruzi Homogenates

**Figure 2:**
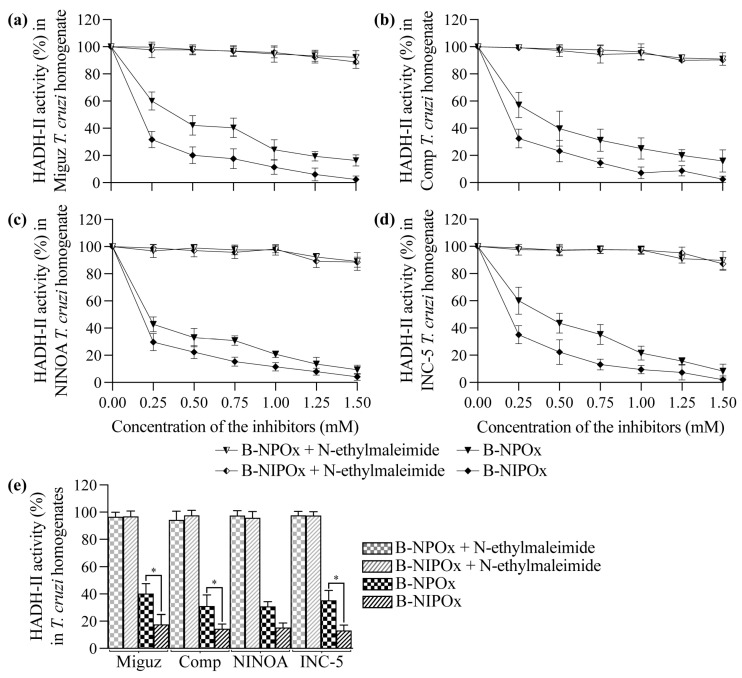
The benzyl ester of N-isopropyl oxamic acid (B-NIPOx) has a better inhibitory effect on the α-hydroxy acid dehydrogenase isozyme II (HADH-II) than the benzyl ester of N-propyl oxamic acid (B-NPOx) in *Trypanosoma cruzi* homogenates. The inhibitory activity of B-NPOx and B-NIPOx over the HADH-II was analyzed with or without 1 mM N-ethylmaleimide in homogenates of (**a**) Miguz, (**b**) Compostela (Comp), (**c**) NINOA or (**d**) INC-5 *T. cruzi* strains, and in the presence of 0.12 mM NADH^+^ + H^+^ and 5 mM α-ketoisocaproate. The inhibitors were evaluated at concentrations of 0.25, 0.50, 0.75, 1.00, 1.25 and 1.50 mM. The HADH-II activity was measured by the changes in the absorption at 340 nm over 5 min and was expressed as a percentage of ΔE 340 nm/min, where the enzymatic activity in absence of inhibitor was considered 100%. (**e**) Asterisks represent statistically significant differences between the HADH-II activity in the presence of 0.75 mM inhibitors (* *p* ≤ 0.05). *n* = 6. One experiment representative of three is shown.

NPOx and NIPOx (Figure 1a) decreased the activity of the purified HADH-II from the Miguz (Figure 1b), Compostela (Figure 1c), NINOA (Figure 1d) and INC-5 (Figure 1e) *T. cruzi* strains. NIPOx is an analog of the main HADH-II substrate (α-ketoisocaproate) [17], and so it causes a greater inhibition of the purified enzyme from the four *T. cruzi* strains at all tested concentrations than NPOx (Figure 1b–f). NIPOx decreased HADH-II activity to less than 20% starting from a concentration of 0.50 mM in all *T. cruzi* strains, while NPOx caused this effect from a concentration of 1.25 mM in the NINOA and INC-5 strains, and from a concentration of 1.50 mM in the Miguz and Compostela strains (Figure 1b–f). The benzyl esters of NPOx and NIPOx did not affect the activity of the purified HADH-II from the four *T. cruzi* strains at any concentration (Figure 1b–f) because the benzyl group interferes with the specificity of the inhibitors for the active site of the enzyme. However, B-NPOx and B-NIPOx decreased the activity of HADH-II in homogenates of the Miguz (Figure 2a), Compostela (Figure 2b), NINOA (Figure 2c) and INC-5 (Figure 2d) *T. cruzi* strains, since the carboxylesterases present in the homogenates can cleave the benzyl esters and release the inhibitors to the medium. This mechanism was confirmed by adding a carboxylesterases inhibitor, N-ethylmaleimide [24], to the homogenates. In the presence of N-ethylmaleimide, B-NPOx and B-NIPOx did not decrease the activity of HADH-II in the homogenates of the four *T. cruzi* strains at all tested concentrations (Figure 2a–e). B-NIPOx presented a higher inhibitory effect than B-NPOx in the four *T. cruzi* strain homogenates at all concentrations (Figure 2a–e). B-NIPOx decreased HADH-II activity in all *T. cruzi* homogenates to less than 20% from a concentration of 0.75 mM, while B-NPOx caused this effect from a concentration of 1.25 mM in the Miguz, NINOA and INC-5 strains, and from a concentration of 1.50 mM in the Compostela strain.

### 2.4. B-NIPOx Had Higher Trypanocidal Activity on Trypanosoma cruzi Epimastigotes and Trypomastigotes than B-NPOx and the Reference Drugs Benznidazole and Nifurtimox

**Figure 3:**
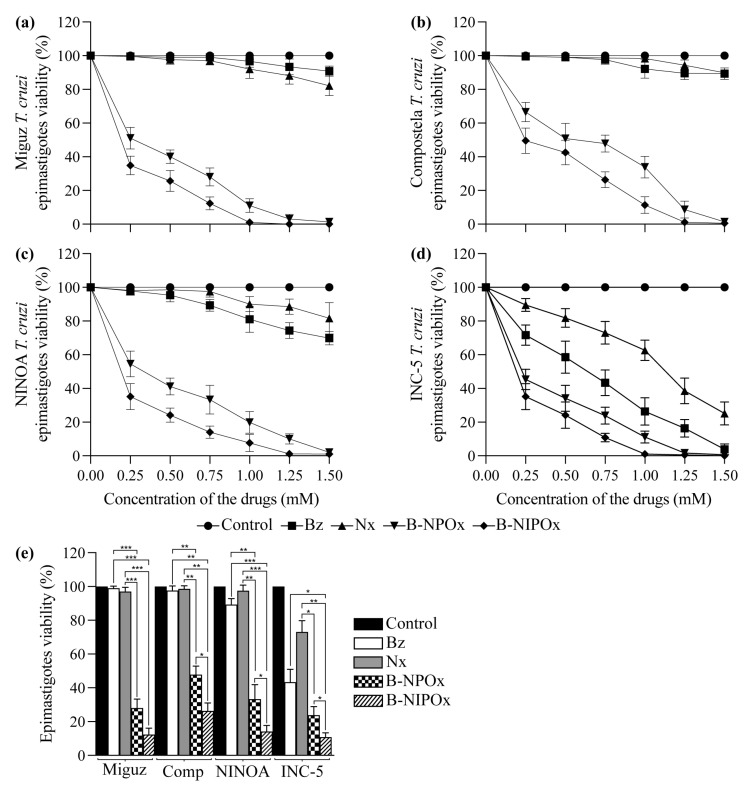
The benzyl ester of N-isopropyl oxamic acid (B-NIPOx) has a higher trypanocidal activity on epimastigotes than the benzyl ester of N-propyl oxamic acid (B-NPOx) and the reference drugs Benznidazole and Nifurtimox. The trypanocidal effect of B-NIPOx was evaluated on epimastigotes of the (**a**) Miguz, (**b**) Compostela (Comp), (**c**) NINOA and (**d**) INC-5 *T. cruzi* strains and compared with the drugs B-NPOx, Benznidazole (Bz) and Nifurtimox (Nx). The drugs were dissolved in 1% DMSO and were evaluated at concentrations of 0.25, 0.50, 0.75, 1.00, 1.25 and 1.50 mM for 60 min at 28 °C in cultures with 1 × 10^6^ epimastigotes/mL. The viability of the epimastigotes was determined by the alamarBlue method; the control, which was set to 100%, represents the viability of the parasites in 1% DMSO. (**e**) Asterisks represent statistically significant differences between the viability of the epimastigotes *T. cruzi* strains treated with 0.75 mM drugs (* *p* ≤ 0.05, ** *p* ≤ 0.01 and *** *p* ≤ 0.001). *n* = 6. One experiment representative of three is shown.

**Figure 4:**
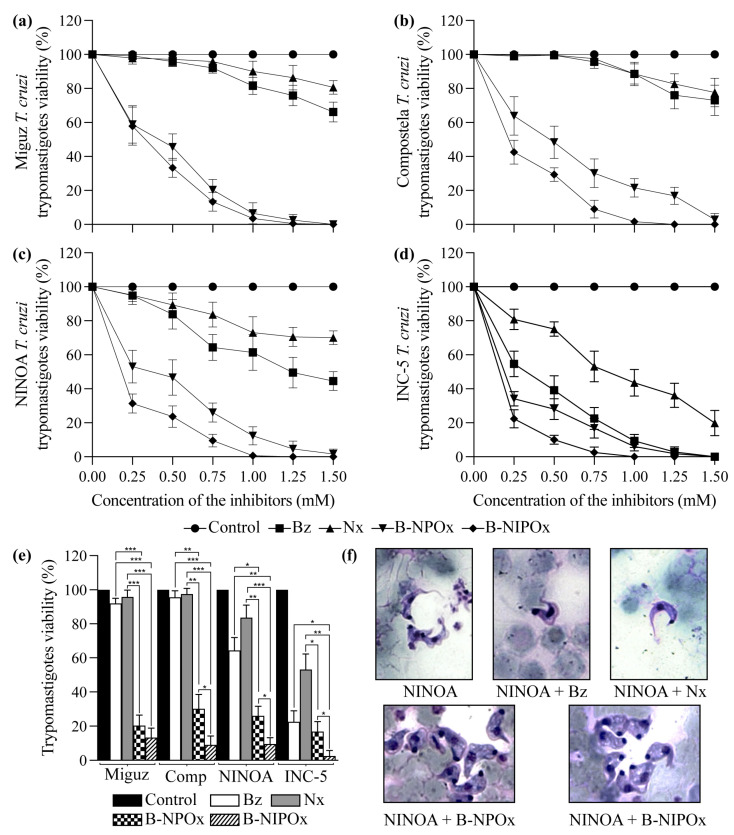
The benzyl ester of N-isopropyl oxamic acid (B-NIPOx) has higher trypanocidal activity on trypomastigotes than the benzyl ester of N-propyl oxamic acid (B-NPOx) and the reference drugs Benznidazole and Nifurtimox and alters the typical morphology of the parasite. The trypanocidal effect of B-NIPOx was evaluated on trypomastigotes of the (**a**) Miguz, (**b**) Compostela (Comp), (**c**) NINOA and (**d**) INC-5 *T. cruzi* strains, and compared with the drugs B-NPOx, Benznidazole (Bz) and Nifurtimox (Nx). The drugs were dissolved in 1% DMSO and were evaluated at concentrations of 0.25, 0.50, 0.75, 1.00, 1.25 and 1.50 mM for 24 h at 4 °C in cultures with 1 × 10^6^ trypomastigotes/mL. The viability of the trypomastigotes was determined by the Filardi and Brener method; the control, which was set to 100%, represents the viability of the parasites in the presence of 1% DMSO. (**e**) Asterisks represent statistically significant differences between the viability of the trypomastigotes of the four *T. cruzi* strains treated with 0.75 mM drugs (* *p* ≤ 0.05, ** *p* ≤ 0.01 and *** *p* ≤ 0.001). *n* = 6. One experiment representative of three is shown. (**f**) 40× photographs of Giemsa-stained blood trypomastigotes.

B-NIPOx and B-NPOx had a higher trypanocidal effect at all concentrations, compared to the reference drugs Benznidazole and Nifurtimox on epimastigotes (Figure 3a–d) and trypomastigotes (Figure 4a–d) of the Miguz, Compostela, NINOA and INC-5 *T. cruzi* strains. At all the tested concentrations, Benznidazole and Nifurtimox did not completely decrease the viability of the epimastigotes (Figure 3a–e) or the trypomastigotes (Figure 4a–e). In fact, the epimastigotes (Figure 3a,b) and trypomastigotes (Figure 4a,b) of the Miguz and Compostela strains were resistant to these drugs at the tested concentrations. B-NIPOx caused the death of all epimastigotes at lower concentrations than NPOx. At a concentration of 1.00 mM, B-NIPOx caused the death of all epimastigotes of the Miguz and INC-5 strains, and at a concentration of 1.25 mM, it caused the death of all epimastigotes of the Compostela and NINOA strains, while B-NPOx caused this effect at a concentration of 1.5 mM in all the strains (Figure 3a–e). The same effect was observed in the trypomastigotes, where B-NIPOx caused the death of all the trypomastigotes from the four strains at a concentration of 1 mM, while B-NPOx caused this effect at a concentration of 1.5 mM (Figure 4a–e). In the presence of 0.75 mM B-NPOx or B-NIPOx, the trypomastigotes of the four *T. cruzi* strains (Figure 4f) strains had an increase in their size and in their number of vacuoles but a decrease in the length of their flagellum. On the other hand, the trypomastigotes treated with Benznidazole or Nifurtimox had no morphological alterations (Figure 4f).

### 2.5. B-NIPOx Was More Effective in Reducing Blood Parasitemia and the Amastigote Nests in Infected Mice than B-NPOx and the Reference Drugs Benznidazole and Nifurtimox

**Figure 5:**
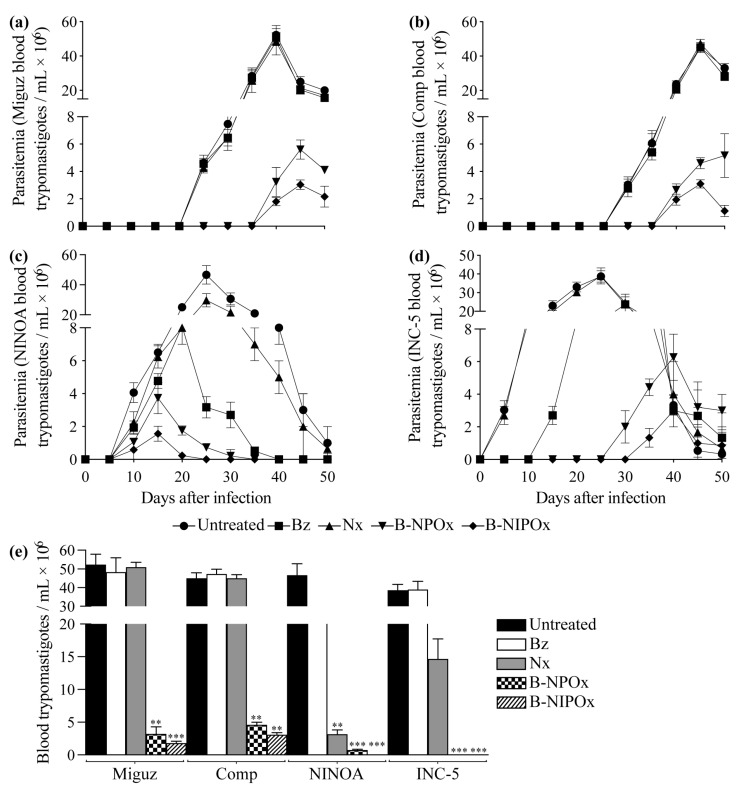
The benzyl ester of N-isopropyl oxamic acid (B-NIPOx) is more effective to reduce the blood parasitemia of mice infected with *Trypanosoma cruzi* than the benzyl ester of N-propyl oxamic acid (B-NPOx) and the reference drugs Benznidazole and Nifurtimox. Parasitemia was evaluated in the blood of mice infected with the (**a**) Miguz, (**b**) Compostela (Comp), (**c**) NINOA or (**d**) INC-5 *T. cruzi* strains. The infected mice were treated with Benznidazole (Bz), Nifurtimox (Nx), B-NPOx or B-NIPOx with daily dosing at 100 mg/Kg for 50 days. Blood samples were collected every 5 days from the tail vein, and the parasitemia was determined with the Filardi and Brener method; the controls were infected and untreated mice. (**e**) Asterisks represent statistically significant differences between the blood parasitemia of mice infected with the *T. cruzi* strains and treated with Bz, Nx, B-NPOx or B-NIPOx, compared to the infected and untreated mice, at the time the untreated mice reach the parasitemia peak (** *p* ≤ 0.01 and *** *p* ≤ 0.001) *n* = 6. One experiment representative of three is shown.

In mice infected with the Miguz *T. cruzi* strain and in mice infected with this strain and treated with Benznidazole or Nifurtimox, the parasitemia was detectable on day 25, with peak parasitemia on day 40; while in mice treated with B-NPOx or B-NIPOx, the parasitemia was detectable on day 40, with peak parasitemia on day 45 (Figure 5a). In mice infected with the Compostela strain and in mice infected with this strain and treated with Benznidazole or Nifurtimox, the parasitemia was detectable on day 30, with peak parasitemia on day 45; while in mice treated with B-NPOx or B-NIPOx, the parasitemia was detectable on day 40, with peak parasitemia on day 50 (B-NPOx) or day 45 (B-NIPOx) (Figure 5b). In mice infected with the NINOA strain and in mice infected with this strain and treated with Benznidazole, Nifurtimox, B-NPOx or B-NIPOx, the parasitemia was detectable on day 10, with peak parasitemia on day 25 (untreated and Nifurtimox), day 20 (Benznidazole) and day 15 (B-NPOx and B-NIPOx) (Figure 5c).

In mice infected with the INC-5 strain, and in mice infected with this strain and treated with Benznidazole, Nifurtimox, B-NPOx or B-NIPOx, the parasitemia was detectable on day 5 (untreated and Nifurtimox), day 15 (Benznidazole), day 30 (B-NPOx) and day 35 (B-NIPOx), with peak parasitemia on day 25 (untreated and Nifurtimox), day 30 (Benznidazole) and day 40 (B-NPOx and B-NIPOx) (Figure 5d). In mice infected with the Miguz, Compostela or INC-5 strains, no statistically significant differences were found in the number of blood trypomastigotes/mL between untreated mice and mice treated with Benznidazole or Nifurtimox. However, statistically significant differences (** *p* ≤ 0.01 and *** *p* ≤ 0.001) were found between untreated mice and mice treated with B-NPOx or B-NIPOx (Figure 5e).

**Figure 6:**
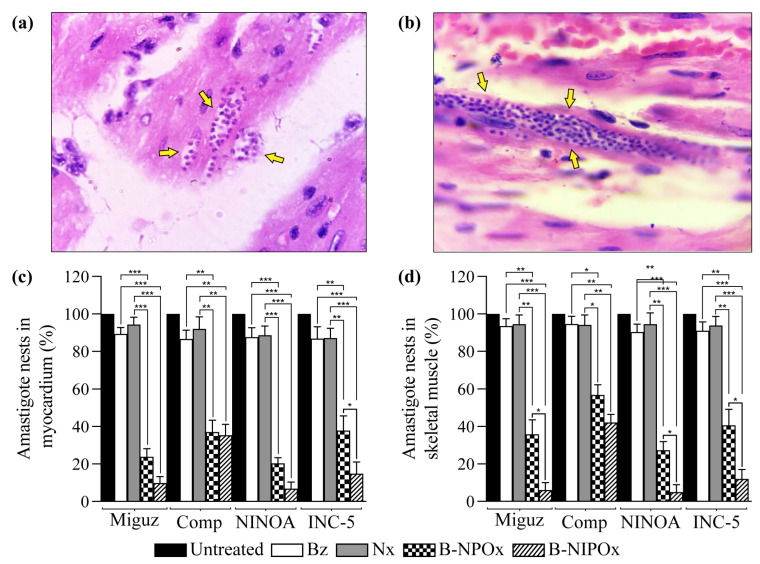
The benzyl ester of N-isopropyl oxamic acid (B-NIPOx) is more effective to reduce the percentage of amastigote nests in the myocardium and in the skeletal muscle of mice infected with *Trypanosoma cruzi* than the benzyl ester of N-propyl oxamic acid (B-NPOx) and the reference drugs Benznidazole and Nifurtimox. Representative photographs (40×) of (**a**) the myocardium and (**b**) the skeletal muscle of CD-1 mice infected with the INC-5 *T. cruzi* strain. The yellow arrows indicate the amastigote nests in the myocardium and the skeletal muscle. The amastigote nests of untreated or treated CD-1 mice, which were infected with the Miguz, Compostela (Comp), NINOA or INC-5 *T. cruzi* strains, were counted in myocardium and skeletal muscle slides stained with H/E. The infected mice were treated with Benznidazole (Bz), Nifurtimox (Nx), B-NPOx or B-NIPOx, with daily dosing at 100 mg/Kg for 50 days. The mean number of amastigote nests in the infected and untreated group was set as 100%. Asterisks represent statistically significant differences between the (%) of amastigote nests in (**c**) the myocardium or (**d**) the skeletal muscle (* *p* ≤ 0.05, ** *p* ≤ 0.01 and *** *p* ≤ 0.001). *n* = 6. One experiment representative of three is shown.

Myocardial (Figure 6a) and skeletal muscle (Figure 6b) sections from mice infected with *T. cruzi* were stained with H/E and analyzed to determine the presence of amastigote nests.

The average number of amastigote nests in infected and untreated mice was set as 100%. The average percentage of amastigote nests in mice infected with any of the four *T. cruzi* strains and treated with Benznidazole or Nifurtimox always remained above 86% in the myocardium (Figure 6c) and above 91% in the skeletal muscle (Figure 6d). In contrast, in infected mice that were treated with B-NPOx, the average percentages of amastigote nests in the myocardium were 23, 37, 20 and 38% for the Miguz, Compostela, NINOA and INC-5 strains, respectively, and in infected mice that were treated with B-NIPOx, the averages percentages were 9, 35, 6, and 14% (Figure 6c). In infected mice that were treated with B-NPOx, the average percentage of amastigote nests in the skeletal muscle was 35, 45, 27 and 40%, respectively, and in infected mice that were treated with B-NIPOx, the average percentages were 6, 42, 4 and 12% (Figure 6d). The reference drugs Benznidazole or Nifurtimox had almost no trypanocidal effect on the amastigotes, which contrasted with the strong trypanocidal effect of B-NPOx and B-NIPOx on this phase of the parasite. B-NIPOx had a higher trypanocidal effect than B-NPOX on amastigote in the skeletal muscle.

### 2.6. B-NIPOx Was More Effective in Preventing Chagasic Enteropathy than B-NPOx and the Reference Drugs Benznidazole and Nifurtimox

**Figure 7:**
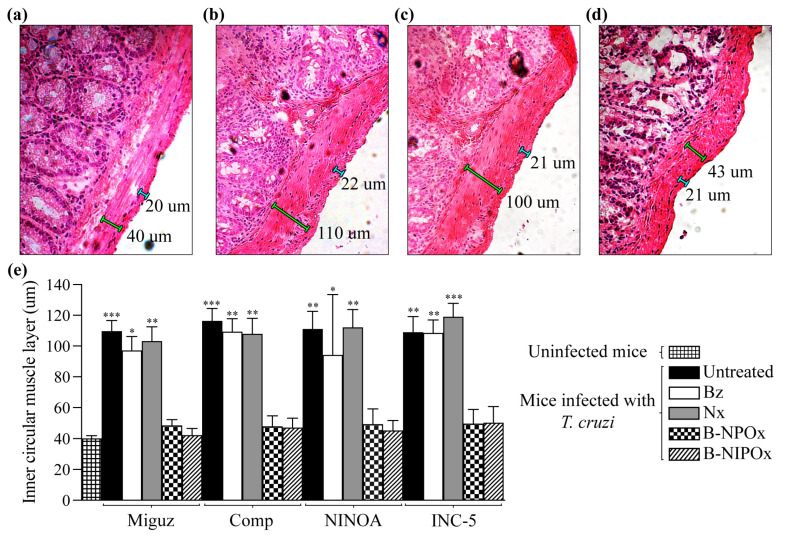
The benzyl ester of N-propyl oxamic acid (B-NPOx) and the benzyl ester of N-isopropyl oxamic acid (B-NIPOx) prevent the thickening of the intestinal inner circular muscle layer of mice infected with *Trypanosoma cruzi*. Representative photographs (40×) of recto-sigmoid large intestinal slides of CD-1 mice, (**a**) uninfected or (**b**–**d**) infected with the INC-5 *T. cruzi* strain, and treated with (**c**) Benznidazole (Bz) or (**d**) B-NIPOx (daily dosing at 100 mg/Kg for 50 days). The widths of the outer longitudinal muscle layer (blue lines) and the inner circular muscle layer (green lines) are indicated. The widths of the inner circular muscle layer of uninfected mice, or of mice infected with the Miguz, Compostela (Comp), NINOA or INC-5 *T. cruzi* strains, were measured. The infected mice were treated with Bz, Nifurtimox (Nx), B-NPOx or B-NIPOx with daily dosing at 100 mg/Kg for 50 days. (**e**) Asterisks represent statistically significant differences between the widths of the inner circular muscle layer of mice infected with the *T. cruzi* strains compared to uninfected mice (* *p* ≤ 0.05, ** *p* ≤ 0.01 and *** *p* ≤ 0.001) *n* = 6. One experiment representative of three is shown.

During the chronic stage of Chagas disease, *T. cruzi* invades the smooth muscle of the digestive system, causing inflammation and thickening of the muscular layers, which trigger the formation of mega syndromes in the esophagus and colon [25]. Therefore, here we analyzed the recto-sigmoid of the large intestine from uninfected mice (Figure 7a), infected mice (Figure 7b) and mice infected and treated mice with Benznidazole (Figure 7c),

Nifurtimox, B-NPOx or B-NIPOx (Figure 7d,e), and measured the widths of the outer longitudinal muscle layer and the inner circular muscle layer (Figure 7a–d). The width of the outer longitudinal muscle layer remained constant in the uninfected, infected, and infected and treated mice, with an average of 21 μm. However, the width of the inner circular muscle layer changed between the mice groups (Figure 7e). The average widths of the inner circular muscle layer of mice infected with the Miguz, Compostela, NINOA or INC-5 strains and treated with Benznidazole were 97, 109, 94 and 108 μm, respectively, and for infected mice treated with Nifurtimox, the average widths were 103, 107, 112 and 119 μm. These widths were not significantly different from those of infected and untreated mice, where the average widths were 109, 116, 111 and 109 μm. In contrast, the average widths of the inner circular muscle layer of infected mice treated with B-NPOx were 48, 49, 50 and 49 μm, respectively, and for infected mice treated with B-NIPOx, the average widths were 42, 47, 45 and 50 μm. These widths were not significantly different from those of uninfected mice, where the average width of this layer was 40 μm (Figure 7e), indicating that B-NPOx and B-NIPOx prevented the thickening of the inner circular muscle layer of the large intestine during *T. cruzi* infection.

**Figure 8:**
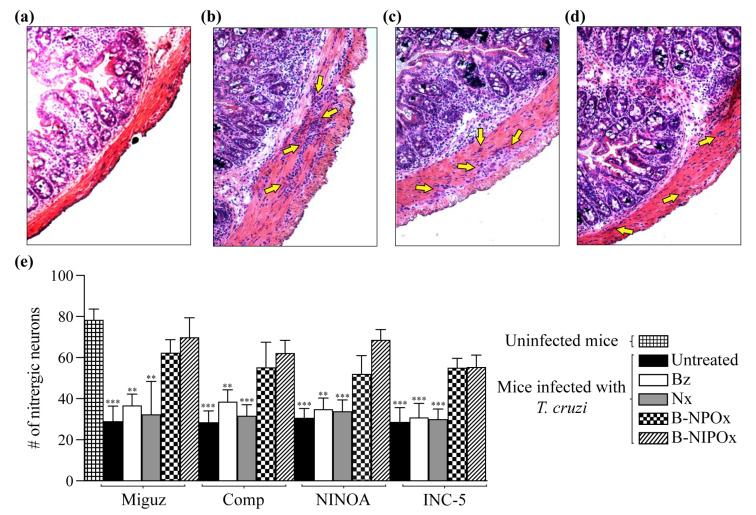
The benzyl ester of N-propyl oxamic acid (B-NPOx) and the benzyl ester of N-isopropyl oxamic acid (B-NIPOx) prevent leukocyte infiltration in the intestinal inner circular muscle layer, and the loss of nitrergic neurons in the intestine of mice infected with *Trypanosoma cruzi*. Representative photographs (40×) of recto-sigmoid large intestinal slides of CD-1 mice, (**a**) uninfected or (**b**–**d**) infected with the NINOA *T. cruzi* strain and treated with (**c**) Benznidazole (Bz) or (**d**) B-NIPOx (daily dosing at 100 mg/Kg for 50 days). The yellow arrows indicate the leukocyte infiltration in the intestinal inner circular muscle layer. The nitrergic neurons of uninfected mice, or of mice infected with the Miguz, Compostela (Comp), NINOA or INC-5 *T. cruzi* strains, were counted in recto-sigmoid large intestinal slides stained with NADPH diaphorase. The infected mice had been treated with Bz, Nifurtimox (Nx), B-NPOx or B-NIPOx with daily dosing at 100 mg/Kg for 50 days. (**e**) Asterisks represent statistically significant differences between the nitrergic neurons of the recto-sigmoid large intestinal of mice infected with the *T. cruzi* strains, compared with the uninfected mice (** *p* ≤ 0.01 and *** *p* ≤ 0.001). *n* = 6. One experiment representative of three is shown.

During Chagasic enteropathy, the intestine muscle layers are infiltrated with leukocytes [26]. Here we analyzed the recto-sigmoid of the large intestine and found that mice infected with the Miguz, Compostela, NINOA or INC-5 *T. cruzi* strains (Figure 8b) had a severe leukocyte infiltrate in the internal circular muscle layer, which is not observed in uninfected mice (Figure 8a) but was present to a lesser extent in infected mice that were treated with Benznidazole (Figure 8c) or Nifurtimox. In mice infected and treated with B-NPOx or with B-NIPOx (Figure 8d), the leukocyte infiltrate is scarce and consists mainly of individual cells. In infected mice treated with Benznidazole or Nifurtimox and in infected and untreated mice, the infiltrating cells form large clusters.

Another feature of Chagasic enteropathy is a massive neuronal loss in the plexuses that conform to the enteric nervous system [27,28]. Here, we counted the number of nitrergic neurons in the recto-sigmoids from uninfected, infected and infected and treated mice. The average numbers of nitrergic neurons in mice infected with the Miguz, Compostela, NINOA and INC-5 strains and treated with Benznidazole were 36, 38, 34 and 33, respectively, and for infected mice treated with Nifurtimox, the average numbers of nitrergic neurons were 32, 31, 33 and 30. These numbers were not significantly different from those of infected and untreated mice, where the average numbers of nitrergic neurons were 29, 28, 30 and 28. In contrast, the average numbers of nitrergic neurons in infected mice treated with B-NPOx were 62, 56, 52 and 55, respectively, and for infected mice treated with B-NIPOx, the average numbers of nitrergic neurons 69, 62, 68 and 57. These numbers were not significantly different from those of uninfected mice, where the average number of nitrergic neurons was 78 (Figure 8e), indicating that B-NPOx and B-NIPOx prevent the loss of nitrergic neurons in the large intestine during *T. cruzi* infection.

## 3. Discussion

More than 100 years have passed since the discovery of Chagas disease but many challenges still remain unresolved. The methods required for the epidemiological control, diagnosis, treatment and prognosis of this disease must be improved. In particular, the efficacy and tolerability profile of the currently available therapeutic agents is far from ideal [29]. Benznidazole and Nifurtimox are the only drugs available for the treatment of Chagas disease; both were released in the 1960s and have undesirable side effects [30], long periods of treatment, low antiparasitic activity in the chronic phase of the disease and the presence of resistant strains [31]. Therefore, the development of new drugs that eliminate the parasite to prevent the progression to organ damage, particularly cardiac and digestive damage, is indispensable.

The metabolism of *T. cruzi* has key differences compared to the metabolism of its mammal host and several of the enzymes that are responsible for these differences were selected as possible therapeutic targets [32]. However, since glycolysis is the main metabolic pathway used by *T. cruzi* to obtain its energy [33], we focused on its inhibition by targeting HADH-II, which participates in NADH + H^+^ reoxidation [20]. In previous works, we developed NPOx and NIPOx [21], which are structural analogs of the HADH-II substrates (α-ketocaproate and α-ketoisocaproate) [20] and were excellent competitive and selective inhibitors of HADH-II. However, they had low trypanocidal activity in in vivo tests because they are polar molecules that do not cross the parasite membrane. Therefore, we synthesized the benzyl ester of NPOx (B-NPOx), which is non-polar and crosses cell membranes, and presents good in vivo trypanocidal activity [22]. In this work, we designed and synthesized B-NIPOx, the benzyl ester of NIPOx, which is structurally analogous to α-ketoisocaproate, the substrate for which HADH-II has the highest affinity [17]. The correct synthesis of B-NIPOx was confirmed by IR, ^1^H-NMR and ^13^C-NMR, where its functional groups were identified. We demonstrated that B-NIPOx had no cytotoxicity on three cell lines, and no toxicity in mice (the LD_50_ value was 2.15 g/Kg of body weight, which indicates that B-NIPOx is not toxic to mice [23]).

B-NIPOx and B-NPOx did not decrease the enzymatic activity of HADH-II purified from four *T. cruzi* strains because the added benzyl groups interfered with the specificity of the inhibitors for the active site of the enzyme. As expected, NIPOx and NPOx decreased the activity of purified HADH-II, and NIPOx was more effective than NPOx since NIPOx is an analog of the enzyme’s main substrate. The benzyl esters were designed to convert the oxamic acids to non-polar drugs that cross the parasite membrane. To determine if B-NIPOx could inhibit HADH-II in vivo, we tested it in *T. cruzi* homogenates, which contain the parasite’s many degradative enzymes, including carboxylesterases [34] that cleave benzyl esters. B-NIPOx decreased HADH-II activity in *T. cruzi* homogenates, more efficiently than B-NPOx. When this experiment was performed in the presence of N-ethylmaleimide, a carboxylesterase inhibitor, neither B-NIPOx nor B-NPOx decreased HADH-II activity, which confirms that cleaving of the benzyl groups by carboxylesterases is required to release the inhibitors NIPOx and NPOx.

Benznidazole and Nifurtimox are effective in the acute phase of Chagas disease but there are many resistant *T. cruzi* strains [35]. We observed that the epimastigotes and trypomastigotes of the Miguz and Compostela strains were highly resistant to the trypanocidal effects of Benznidazole and Nifurtimox, while the NINOA and INC-5 strains were killed by these drugs. However, B-NIPOx had higher trypanocidal activity than B-NPOx and the reference drugs on the four *T. cruzi* strains at lower concentrations. Since *T. cruzi* is a parasite, it depends on the carbon sources present in the host for its energy metabolism, and glucose is its preferred carbon source [36]. The strong trypanocidal effects of B-NIPOx and B-NPOx could be attributed not only to HADH-II inhibition but also to the release of benzyl alcohol, which causes changes in cell membrane fluidity and has direct antimicrobial activity [37]. The trypomastigotes of the four *T. cruzi* strains showed significant morphological changes, with increased size and numbers of vacuoles, when they were treated with B-NIPOx or B-NPOx, but not when they were treated with Benznidazole or Nifurtimox, which could reflect the accumulation of benzyl alcohol in the former cases.

To further characterize the effects of B-NIPOX, we used an in vivo model of *T. cruzi* infection. In our experimental design, mice were infected with blood trypomastigotes from each *T. cruzi* strain on day 0, and then received a daily dose of Benznidazole, Nifurtimox, B-NPOx or B-NIPOx for 50 days. We observed that B-NIPOx was more effective than Benznidazole and Nifurtimox to reduce blood parasitemia and amastigote nests and that, in contrast to these drugs, it prevented the development of Chagasic enteropathy. Since we demonstrated that B-NIPOx has in vitro trypanocidal activity on trypomastigotes, a likely explanation for these observations is that B-NIPOx (but not Benznidazole or Nifurtimox) prevented, or at least reduced, the establishment of the infection with the four *T. cruzi* strains. Thus, the clearing of blood trypomastigotes would prevent or reduce tissue invasion, leading to decreased numbers of amastigote nests (amastigotes are the replicative intracellular form of the parasite, and characterize the chronic phase of Chagas disease). However, it is also possible that B-NIPOx has a direct trypanocidal effect on tissue amastigotes since amastigotes also use glucose as a carbon source [38]. To test this hypothesis, B-NIPOx should be administered to mice after the chronic phase of the infection is established. Another interesting possibility would be to test B-NIPOx in chronically infected mice to assess its impact on congenital *T. cruzi* transmission.

In the chronic phase of Chagas disease, parasite persistence and chronic inflammation lead to functional disorders in the digestive tract, with denervation of the autonomic nervous system that leads to motor incoordination, as well as alterations in the muscle layers [39]. These events trigger the Chagasic enteropathy characterized by intestinal mega syndromes. B-NIPOx and B-NPOx prevented the thickening of the intestinal inner circular muscle layer of mice infected with the four *T. cruzi* strains. In contrast, Benznidazole and Nifurtimox did not prevent the thickening of this intestinal layer in infected mice. Chagasic enteropathy involves leukocyte infiltration in the enteric plexuses, which induces fibrosis [40]. B-NIPOx and B-NPOx prevented leukocyte infiltration in the intestinal inner circular muscle layer of mice infected with the four *T. cruzi* strains, while Benznidazole and Nifurtimox did not prevent this leukocyte infiltration. Another characteristic of Chagasic enteropathy is the degeneration of nitrergic neurons, which are located in the intestinal wall, are organized in ganglia and are interconnected by nerve fibers that constitute the myenteric and submucosal plexuses [25]. In mice infected with the four *T. cruzi* strains and treated with B-NIPOx or B-NPOx, the numbers of nitrergic neurons were almost the same as in uninfected mice, which is important, since muscle hypertrophy and loss of organ function are associated with the loss of these neurons [40]. In contrast, in infected mice that were treated with Benznidazole or Nifurtimox, the numbers of nitrergic neurons decreased significantly compared to uninfected mice.

An advantage of our in vivo experimental design is that it allows us to evaluate, in a relatively short time, the amastigote nests, the thickening of intestinal muscle layers, the loss of nitrergic neurons and the intestinal leukocyte infiltration, which are characteristics of Chagasic enteropathy. These characteristics take months and even years to develop in other mouse models, making drug-testing experiments time-consuming and expensive [41]. In Chagas disease, it is still considered that mouse models are the most appropriate and well-understood system for the screening of novel therapeutics [42] since they can predict the efficacy of new drugs in humans [41]. Therefore, once promising drugs were identified in our model, they could then be analyzed with other experimental designs to test their effectiveness in the acute and chronic phases of Chagas disease. These experimental designs would reflect the situation of *T. cruzi*-infected patients that receive pharmacological treatments, which our current study design does not reflect. Our results provide evidence that B-NIPOx has in vitro and in vivo trypanocidal activity, and that it prevents the development of Chagasic enteropathy in mice, indicating that it is a promising drug candidate for the treatment of Chagas disease.

## 4. Materials and Methods

### 4.1. Ethics Statement

The mice studies were carried out in the National School of Biological Science of the National Polytechnic Institute, in accordance with the principles of the “Guide for the Care and Use of Laboratory Animals” of the US National Institutes of Health [43]. This protocol (CEI-ENCB-025/2014) was approved by the Bioethics Committee of our institution. Six-week-old male CD-1 mice (25–30 g) were used for all the experiments.

### 4.2. Chemicals

α-ketoisocaproate sodium salt, N-ethylmaleimide and NADH + H^+^ were obtained from Sigma Aldrich (St. Louis, MO, USA). Benznidazole and Nifurtimox were obtained from Roche (Basel, Switzerland) and Bayer (Munich, Germany), respectively. NPOx, B-NPOx and NIPOx were synthesized as previously described [21,22].

### 4.3. Synthesis of the Benzyl Ester of the N-Isopropyl Oxamic Acid (B-NIPOx)

A solution of isopropylamine (4.3 mL, 0.1 mol) (Sigma Aldrich) was dissolved in 50 mL of ethyl ether (Sigma Aldrich). This solution was added dropwise and with permanent stirring at 4 °C for 2 h to a solution prepared with 12.5 g of dibenzyloxalate (Sigma Aldrich), previously dissolved in 200 mL of ethyl ether, and 50 mL of chloroform (Sigma Aldrich). The reaction mixture was then warmed to room temperature and stirred overnight. The benzyl alcohol produced during the reaction was removed by distillation at 50–60 °C/1 mmHg, which caused the product B-NIPOx to begin to crystallize in the system. The crystals were vacuum filtered and placed in chloroform. Finally, the crystals were purified by recrystallization from chloroform to give 13.3 g of the desired product in an overall yield of 93.21%. B-NIPOx had a melting point of 50–53 °C.

The final product was analyzed by IR (ATR) (Perkin Elmer GX spectrometer; Perkin Elmer, Waltham, MA, USA), ^1^H-NMR (Varian VNMRS 500 spectrometer; Varian Associates, Palo Alto, CA, USA) and ^13^C-NMR (Varian VNMRS 500 spectrometer).

### 4.4. Evaluation of the Cytotoxicity and Mice Toxicity of B-NIPOx

The cytotoxicity of B-NIPOx was evaluated with the alamarBlue (Invitrogen, Life Technologies, Sacramento, CA, USA) method in Vero, C-33A and HEK293 cell lines. HEK293 cells were purchased from InvivoGen (San Diego, CA, USA), while Vero and C-33A were from the American Type Culture Collection (Manassas, VA, USA). Prior to the assay, 1 × 10^3^ cells in DMEM medium (Gibco, Grand Island, NY, USA) were plated on 24-well plates (Costar Co., Cambridge, MA, USA) and incubated in a humidified 5% CO_2_ incubator (ThermoFisher Scientific, Waltham, MA, USA) at 37 °C, until 80% of confluence. Subsequently, B-NIPOx, dissolved with 1% DMSO (Sigma Aldrich), was added to a final concentration of 1, 3, 5, 10, 50 and 100 mM; each drug concentration was analyzed 6 times. After 24 h of incubation, 10% of alamarBlue was added to each well, and the fluorescence was measured 24, 48, and 72 h later in a spectrofluorometer (Perkin Elmer LS 55). The cell viability (%) relative to control wells (cultured in medium with 1% DMSO) was calculated (% = [fluorescence_test_/fluorescence_control_]100).

The median lethal dose (LD_50_) value of B-NIPOx was determined in CD-1 mice, according to the established requirements [23]. B-NIPOx was administered orally as solution in 5% arabic gum in water at doses of 1.50–3.00 g/kg of body weight on a daily basis for 50 days. The mice were then sacrificed, and autopsies were performed. Thin sections of heart, kidneys, lungs, spleens, livers and intestine were analyzed for histological alterations.

### 4.5. Trypanosoma cruzi Strains

The *T. cruzi* strains used in this study (Miguz, Compostela, NINOA and INC-5) correspond to the prevalent discrete typing unit (DTU) classification designed as TcI [44,45]. The *T. cruzi* strains were preserved in triatomine bugs (*Meccus longipennis*) and by serial passages in CD-1 mice [46] in the Helminthology Laboratory of the Parasitology Department of the National School of Biological Sciences.

### 4.6. Preparation of Trypanosoma cruzi Homogenates, and Purification of α-Hydroxy Acid Dehydrogenase Isozyme II

Suspensions of the four *T. cruzi* strains (3 × 10^6^ trypomastigotes/mL) were lysed by three cycles of freezing in liquid nitrogen and thawing at room temperature. The disruption of the parasites was monitored by microscopy of the resulting homogenates, with the Axio Observer D1 microscope (Carl Zeiss, Thornwood, NY, USA). Protease inhibitors were not used to obtain the homogenates, because they could inhibit the aliphatic and aromatic carboxyl esterase activities described in *T. cruzi* [47,48]. The homogenates were frozen at −20 °C until the HADH-II activity analysis. Prior the HADH-II enzymatic assays, the homogenates were thawed at room temperature and centrifuged at 1200× *g* for 20 min at 4 °C. The HADH-II was purified as previously described [20].

### 4.7. Quantification of α-Hydroxy Acid Dehydrogenase Isozyme II Activity

The HADH-II activity was spectrophotometrically measured in a Perkin Elmer GX spectrometer (Perkin Elmer). HADH-II oxidizes NADH + H^+^ (λ = 340 nm, ε = 6220 M^−1^ cm^−1^) with α-ketoisocaproate as substrate [20]. Each assay mixture contained phosphate-buffered saline (PBS) pH 7.4 (Gibco), 0.12 mM NADH + H^+^, 5 mM α-ketoisocaproate neutral sodium salt, and the purified HADH-II in a final volume of 3 mL. The assay mixtures were incubated at 37 °C, and changes in the absorption at 340 nm were recorded over a 5 min period. The activity of HADH-II was subsequently expressed as the percentage of ΔE 340 nm/min. The activity of HADH-II was also measured in the presence of 0.25, 0.50, 0.75, 1.00, 1.25 and 1.50 mM of NPOx, NIPOx, B-NPOx or B-NIPOx, where the activity in the absence of an inhibitor was considered 100%. In addition, the activity of HADH-II in the homogenates of each of the four *T. cruzi* strains was measured, using the whole homogenates instead of the purified HADH-II.

### 4.8. Trypanocidal Activity of B-NIPOx on Trypanosoma cruzi Epimastigotes

Epimastigotes of the Miguz, Compostela, NINOA and INC-5 *T. cruzi* strains were cultured as previously described [22], and their viability was evaluated in the presence of Benznidazole, Nifurtimox, B-NPOx or B-NIPOx, when the parasite cultures were in logarithmic phase [20]. The epimastigotes were harvested and placed in each well of a 96-well plate (Costar), with a concentration of 1 × 10^6^ epimastigotes/mL in BHI medium (Sigma Aldrich) supplemented with 10% bovine fetal serum (BFS) (Gibco) and 0.1% penicillin/streptomycin antibiotic (Gibco, 100 U/mL–100 mg/mL). Subsequently, the drugs, dissolved with 1% DMSO, were added to a final concentration of 0.25, 0.50, 0.75, 1.00, 1.25 and 1.50 mM and the cultures were incubated at 28 °C for 60 min. Each drug concentration was analyzed 6 times. The samples were stained with alamarBlue and the fluorescence of each well was measured using a Perkin Elmer LS 55 spectrofluorometer (Perking Elmer). Epimastigote viability (%) was measured relative to the control wells (epimastigotes with 1% DMSO but without drugs), according to the following calculation: Epimastigote viability (%) = (fluorescence_test_ × 100%)/fluorescence_control_. The fluorescence in the control group was taken as 100%.

### 4.9. Trypanocidal Activity of B-NIPOx on Trypanosoma cruzi Blood Trypomastigotes

CD-1 mice (25–30 g) were parasitized with 1 × 10^6^ blood trypomastigotes/mL from mice infected with the Miguz, Compostela, NINOA or INC-5 *T. cruzi* strains. Parasitemia was monitored, and once it reached its peak, blood was obtained by cardiac puncture and the trypomastigote concentration was adjusted to 2 × 10^6^ trypomastigotes/mL with PBS pH 7.2 (Gibco). Subsequently, 1 × 10^6^ blood trypomastigotes were placed in each well of a 96-well plate (Costar). The drugs Benznidazole, Nifurtimox, B-NPOx or B-NIPOx, dissolved with 1% DMSO, were placed in the wells at a final concentration of 0.25, 0.50, 0.75, 1.00, 1.25 or 1.50 mM. Trypomastigotes with 1% DMSO were used as a negative control. The plates were incubated at 4 °C for 24 h. After this incubation, 5 µL aliquots were taken from each well and placed on a slide, where viable trypomastigotes were counted by the Filardi and Brener method, in which mobile parasites are counted in 25 microscope fields at 40× [49], using an Axio Observer D1 microscope (Carl Zeiss). The results were expressed as trypomastigote viability (%); the negative control, which was set to 100%, represents the viability of the parasites in the presence of 1% DMSO. Giemsa staining of the trypomastigotes from each condition was performed, in order to search for morphological changes induced by the drugs at different concentrations. The slides were also analyzed in the Axio Observer D1 microscope (Carl Zeiss) at 40×.

### 4.10. Effect of B-NIPOx on the Parasitaemia and the Amastigote Nests of Trypanosoma cruzi-Infected Mice

Four groups of CD-1 mice (20–30 g) were parasitized with 1 × 10^6^ blood trypomastigotes/mL of the Miguz, Compostela, NINOA or INC-5 *T. cruzi* strains, respectively. Each group had 5 subgroups with 6 mice. One of these subgroups remained untreated; while the other four were treated with Benznidazole, Nifurtimox, B-NPOx or B-NIPOx. The drugs were orally administered as solutions in 5% Arabic gum in water at a dose of 100 mg/kg every day, over a period of 50 days [20]; the first dose was given 24 h after the infection. Every five days, 5 µL of blood was collected from the tail vein of the infected mice (Supplementary Figure S1). The blood was diluted 1:10 (*v*/*v*) with a saturated solution of ammonium chloride (Sigma Aldrich) [50] and placed in a Neubauer hemocytometer. The levels of parasitemia were determined by the Filardi and Brener method [49], starting 24 h after infection, using an Axio Observer D1 microscope (Carl Zeiss) at 40×.

On day 50, mice were euthanized and tissue samples from the heart myocardium and the skeleton muscle from the legs were used to evaluate the activity of B-NIPOx towards amastigote nests. The tissue samples were fixed with formaldehyde (Polysciences, Warrington, PA, USA), dehydrated and embedded in paraffin (Polysciences). Sections of the tissue samples (3 µm thick) were stained with hematoxylin and eosin (H/E) and analyzed in the Axio Observer D1 microscope (Carl Zeiss) at 40×. Six slides from each mouse were examined to quantify the number of amastigote nests. The mean number of amastigote nests of the untreated subgroups was taken as 100%.

### 4.11. Effect of B-NIPOx on the Chagasic Enteropathy of Trypanosoma cruzi-Infected Mice

Tissue samples from the recto-sigmoid of the large intestines from uninfected mice, from mice infected with the Miguz, Compostela, NINOA or INC-5 *T. cruzi* strains, and from infected mice that were treated with Benznidazole, Nifurtimox, B-NPOx or B-NIPOx (as described in the previous section), were used to evaluate the effect of B-NIPOx on Chagasic enteropathy. The tissue samples were embedded in Tissue-Tek (Sakura Finetek, Torrance, CA, USA), frozen, and stored at −70 °C. Tissues were cut in 6 μm sections using a cryostat (Leica 1900 CM, Wetzlar, DE, Germany). Tissue sections were mounted on poly-L-Lysine-coated slides, fixed in 2% formaldehyde for 1 h at room temperature and washed in PBS. Six slides from each mouse were stained with H/E or with the NADPH-diaphorase technique and analyzed in the Axio Observer D1 microscope (Carl Zeiss). The outer longitudinal muscle layer and the inner circular muscle layer were measured (μm) in the slides stained with H/E. Leukocyte infiltration of the intestinal inner circular muscle layer was also assessed in these slides. The nitrergic neurons were counted in the slides stained with NADPH-diaphorase.

### 4.12. Statistical Analysis

Statistical analysis was performed using GraphPad Prism version 9 (GraphPad, San Diego, CA, USA). For the enzyme activity analysis, the comparison of the enzyme inhibition by NPOx, B-NPOx, NIPOx and B-NIPOx was performed with the Mann–Whitney U test. To compare the trypanocidal activity of Benznidazole, Nifurtimox, B-NPOx and B-NIPOx on epimastigotes, trypomastigotes and amastigotes of the Miguz, Compostela, NINOA or INC-5 *T. cruzi* strains, data were analyzed with a one-way ANOVA followed by Tukey’s multiple comparisons test. To determine the effect of Benznidazole, Nifurtimox, B-NPOx and B-NIPOx on blood parasitemia and on Chagasic enteropathy, the number of blood trypomastigotes/mL, the widths of the inner circular muscle layer and the number of nitrergic neurons were analyzed with the Kruskal–Wallis test with Dunn’s post-test. The results are presented as means ± standard deviations in all cases.

## 5. Conclusions

The new drug B-NIPOx did not cause cytotoxicity in the tested cell lines or toxicity in mice, and it is a more effective inhibitor of HADH-II in *T. cruzi* homogenates than B-NPOx. B-NIPOx also had a higher trypanocidal activity on epimastigotes and on trypomastigotes of four *T. cruzi* strains compared to the drug B-NPOx and also to the reference drugs Benznidazole and Nifurtimox. B-NIPOx was more effective than B-NPOx, Benznidazole and Nifurtimox to reduce blood parasitemia and muscle amastigotes in infected mice. In addition, B-NIPOx prevented the thickening of the inner circular muscle layer of the large intestine of these mice, as well as its infiltration by leukocytes and its loss of nitrergic neurons, which are characteristic events of Chagasic enteropathy. Therefore, B-NIPOx is a strong candidate for further studies that can lead to its development as an alternative drug for the treatment of Chagas disease.

## Data Availability

The data presented in this study are available on request from the corresponding authors.

## Supplementary Materials

The following supporting information can be downloaded at: https://www.mdpi.com/article/10.3390/ijms24010333/s1.
