# Supplementary figures and images for: Effect of B-NIPOx in Experimental Trypanosoma cruzi Infection in Mice
Source: Int J Mol Sci. 2022 Dec 25;24(1):333. doi: 10.3390/ijms24010333 (PMC9820238; doi:10.3390/ijms24010333)

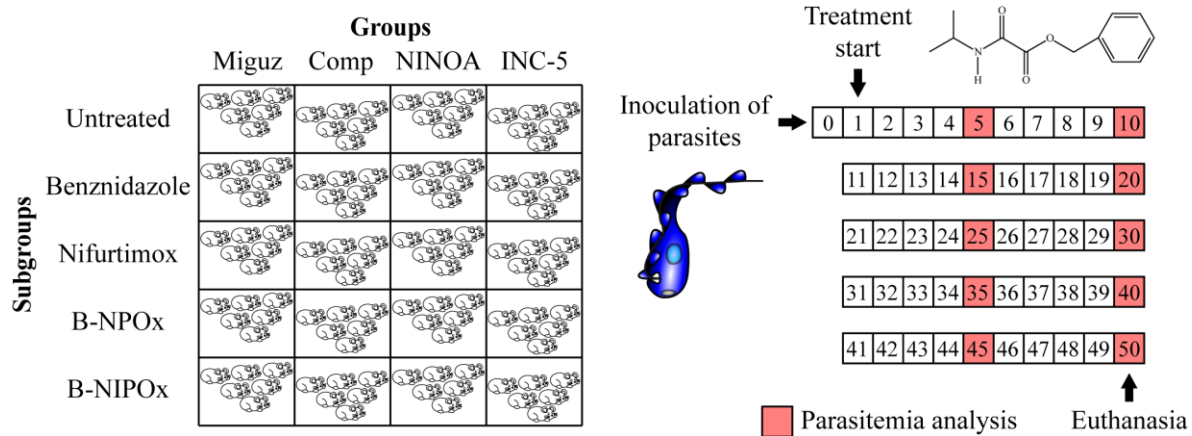

Figure S1: Experimental design in mice

Supplement: Supplementary file 1 [file ijms-24-00333-s001.zip › ijms-2102629-supplementary.pdf]
